# Fertility outcomes following obstetric fistula repair: a prospective cohort study

**DOI:** 10.1186/s12978-017-0415-1

**Published:** 2017-11-28

**Authors:** Dawn M. Kopp, Jeffrey Wilkinson, Angela Bengtson, Ennet Chipungu, Rachel J. Pope, Margaret Moyo, Jennifer H. Tang

**Affiliations:** 1 UNC Project-Malawi, Lilongwe, Malawi; 2 UNC Department of Obstetrics & Gynecology, Chapel Hill, NC USA; 3 UNC Department of Epidemiology, Chapel Hill, NC USA; 4 Fistula Care Center, Lilongwe, Malawi; 5 Malawi College of Medicine Department of Obstetrics & Gynaecology, Blantyre, Malawi; 6 Baylor College of Medicine Department of Obstetrics & Gynecology, Scurlock Tower, 1 Baylor Plaza, Houston, TX 77030 USA

**Keywords:** Obstetric fistula, Fertility, Pregnancy, Amenorrhea, Sexual function, Contraception, Family planning, Malawi, Africa

## Abstract

**Background:**

Obstetric fistula (OF) is a maternal morbidity associated with high rates of stillbirth, amenorrhea, and sexual dysfunction. Limited data exists on the reproductive outcomes of women in the years following a fistula repair. The objective of this study is to describe the fertility outcomes and family planning practices in a population of Malawian women 1–4 years after fistula repair.

**Methods:**

Women who had enrolled into a clinical database of OF patients and undergone OF repair between January 1, 2012 and July 31, 2014 were recruited and enrolled to complete a home-based survey of their demographic and reproductive health data 1–4 years after their repair. Pregnancy, amenorrhea, and sexual function were described using frequency analysis, and we compared antimüllerian hormone (AMH) concentrations between women with menses or pregnancy with women with amenorrhea or no pregnancy using Wilcoxon rank sum tests.

**Results:**

Of 297 women with a prior OF repair, 148 had reproductive potential and were included in this analysis. Overall 30 women of these women (21%) became pregnant since their fistula repair, with most pregnancies ending with cesarean delivery. Of the 32 women who were amenorrheic at the time of repair, 25 (78.1%) had resumption of menses. Only 11 (8.6%) of sexually active women reported dyspareunia, and among women who were not trying to conceive, 53.1% were currently using a method of family planning. No significant differences were found in AMH concentrations between those who were pregnant or had menses versus those without pregnancy or menses, respectively.

**Conclusions:**

In this long-term follow-up study of women after OF repair, many women were able to achieve a pregnancy with a live birth, have normal menses, be sexually active, and access contraception. These achievements will further assist a population of women whose reintegration and restoration of dignity is closely tied to their ability to achieve their reproductive goals.

**Trial registration:**

ClinicalTrials.gov Identifier: NCT02685878.

## Plain English summary

Obstetric fistula (OF) is a birth injury that causes leakage of urine, stool, or both that occurs most frequently in low-income countries. Surgical repair of OF is possible, but there is little data on the reproductive outcomes of women in the years following OF repair. This study describes the fertility outcomes and family planning practices in a population of women in the years following OF repair. Women who had an OF repair in the past 1–4 years were recruited and enrolled to complete a home-based survey of demographic and reproductive health data since their repair. Of the 297 women enrolled with a prior OF repair, 148 were determined to be fertile and included in this analysis. Thirty-one pregnancies since fistula repair were reported among 30 women, with the majority ending with cesarean delivery. Most women who had no menses at the time of repair had resumption of menses at the time of the follow-up survey. Few sexually active women reported pain with sex, and many women accessed effective methods of contraception. In this long-term follow-up study of women after OF repair, many women were able to achieve a pregnancy with a live birth, have normal menses, be sexually active, and access contraception. These achievements will further assist a population of women whose reintegration and restoration of dignity is closely tied to their ability to achieve their reproductive goals.

## Background

Obstetric fistula (OF), which includes both vesicovaginal fistula and rectovaginal fistula, is one of the most debilitating and devastating causes of maternal morbidity in low-income countries. Often the consequence of prolonged and obstructed labor that results in a stillbirth, OF leads to a constant leakage of urine (in a vesicovaginal fistula or VVF), feces (in a rectovaginal fistula or RVF), or both (a combined VVF/RVF), which then often leads to divorce and social isolation [1]. Though surgical repair is possible for many women, the social and reproductive consequences of OF may persist. A full recovery after fistula repair should involve more than simply regaining continence. As Dr. Coetze recognized in his treatment of fistula patients 50 years ago, “For a 100% cure of a patient with vesicovaginal fistula, the following conditions must be fully satisfied: 1) the patient should have compete continence; 2) no stress incontinence should be present; 3) dyspareunia should not occur; 4) traumatic amenorrhea should not occur; and 5) the patient should be able to bear children” [2].

Though not the primary goal of fistula repair, for many women, a return of reproductive capacity is essential to successful reintegration into their communities after surgery [3]. In a recent qualitative study of women 1–2 years after OF repair, 45% of these women desired to have additional children [4]. Reported pregnancy rates in women with a repaired OF range between 10 and 20% [4–7], but this data is limited to small studies that did not evaluate the childbearing potential of the women interviewed. To promote optimal healing and prevent fistula recurrence, women are typically counseled to wait at least 12 months after fistula repair to conceive and then have an scheduled cesarean delivery for all future pregnancies [8]. However, it is unknown how many women are able to adhere to this advice or the outcomes of women who do not adhere. Understanding the fertility outcomes of women who have undergone an OF repair is important in counseling women who are undergoing OF repair and addressing needs after repair.

Amenorrhea may impact the fertility of women after fistula repair since amenorrheic women are unlikely to be ovulating. In women presenting for fistula repair, 20–40% of women have unexplained amenorrhea [6, 7, 9, 10]. Some studies suggest that a subset of these women may resume menses after repair, but data on how frequently this occurs is limited [6, 7]. Additionally, vaginal stenosis is a common sequela of OF [11]. This may not improve after fistula repair and could potentially worsen, impacting sexual function and the ability of a couple to have vaginal intercourse and attempt to conceive [1]. Hormone markers have been shown to predict fertility and amenorrhea [12, 13] and may be useful predictors in women undergoing obstetric fistula repair. Antimüllerian hormone (AMH) has been shown to be a particularly useful predictor because it measures ovarian reserve and unlike other hormones, is not affected by the menstrual cycle.

Finally, though many women desire to become pregnant after OF repair, others may wish to delay or limit their childbearing. In a recent qualitative study, 10% of women who underwent OF repair did not desire future fertility and were interested in accessing long-term and permanent methods of family planning [4]. Therefore, our study objective was to evaluate this population’s long-term fertility desires, outcomes, and family planning practices, so that we can better assist them in achieving their reproductive goals.

## Methods

### Study setting

This study recruited women who had undergone OF repair at the Freedom from Fistula Foundation Fistula Care Centre at Bwaila Hospital in Lilongwe, Malawi. The Fistula Centre receives referrals from all regions of Malawi, as well as western Mozambique and eastern Zambia. Women presenting to the Fistula Care Centre with a confirmed OF are consented for enrollment into a clinical database that includes demographic data, physical exam findings, surgical procedures, post-operative findings (including a post-operative 1-h pad test prior to discharge), and information from three follow-up visits (at months 1, 3, and 12) to the Fistula Care Centre in the first year after repair.

### Study population

Women were eligible for recruitment for this long-term follow-up study if they: (1) had a history of OF repair at the Fistula Care Centre between January 1, 2012 and July 31, 2014 and were enrolled in the database (2) spoke Chichewa (the local language) or English fluently, (3) were age 18 years or above, (4) were currently living in districts in Malawi within 4 h drive of the Fistula Care Centre by motorcycle and (5) were alive at the time of recruitment for this follow-up study. Eligible women identified from the clinical database were traced, recruited, consented, and enrolled in their homes during a visit from a non-medical staff member. We elected to trace women and interview them in their home villages due to the relatively low proportion that return to the Fistula Care Centre for follow-up after their repair (only 20% return for their 12-month follow-up visits).

Women who were traced provided informed consent and completed a survey of demographic, obstetric and gynecologic history, human immunodeficiency virus (HIV) status and testing history and validated measures of quality of life and depressive symptoms [14, 15]. Some of the women traced had not completed any clinical follow-up since their repair. Ethical approval was obtained from the National Health Sciences Research Committee of Malawi (protocol #15/5/1428) and the University of North Carolina School of Medicine Institutional Review Board (Study # 15–0972). The research protocol was registered on clinicaltrials.gov (Identifier: NCT02685878). Trained research assistants double-entered and compared the data using REDCap (Research Electronic Data Capture, NC) [16].

### Variables

Participants’ demographic information, reproductive information, and HIV testing were self-reported. A convenience subset of women included in this analysis had had pelvic ultrasonography performed and hormone markers drawn (AMH, follicle-stimulating hormone [FSH], and estradiol at the time of fistula repair, as a part of another study) [10]. Blood samples were sent to the UNC Project-Malawi Laboratory, where they were centrifuged. Serum was then aliquoted into 1.0 mL cryovials and stored in cryoboxes at −80°C until they were shipped in batches on dry ice to the University of Southern California Reproductive Endocrinology Research Lab. AMH was measured primarily by use of the Ultrasensitive AMH ELISA kit (Ansh Labs, Webster, TX). The picoAMH ELISA kit (Ansh Labs) was used when AMH values were below the limit of detection (<0.07 ng/ml) of the Ultrasensitive ELISA. The limit of detection of the picoAMH ELISA is 0.003 ng/ml. FSH and estradiol was measured by direct immunoassay on the Immulite analyzer (Siemens Healthcare Diagnostics, Deerfield, IL). Hypergonadotropic gonadism was defined as FSH ≥10.0 mIU/ml and Estradiol <20.0 pg/ml.

Reproductive potential was determined after excluding women who were postmenopausal by self-report as their reason for amenorrhea or 50 years or older at the time of the study. Women were also excluded if they had previously undergone hysterectomyor bilateral tubal ligation (in Malawi, bilateral tubal ligations are irreversible, so women who had undergone this surgery could not become pregnant again).

### Analytic methods

Pregnancy, amenorrhea, and sexual function were described using frequency analysis, and Kaplan-Meier estimates were used to calculate the incidence rate for pregnancy. Wilcoxon rank sum tests and linear regression models adjusted for age were used to compare AMH concentrations between: 1) women who became pregnant versus those who did not become pregnant, and 2) women had menses resume and those who did not resume menses. All data were analyzed using Stata Version 13.0 (StataCorp, College Station, TX).

## Results

### Study population

**Fig. 1:**
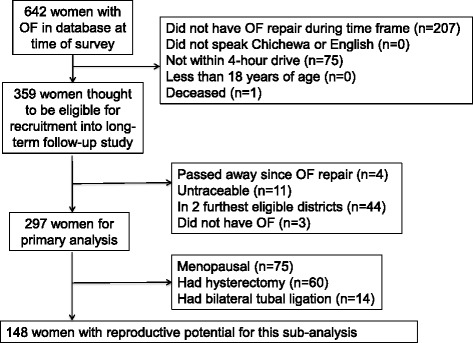
Flow diagram for recruitment and enrollment into study

We identified 359 women who met our eligibility criteria. Four of these women were excluded as they had passed away since their repair, and 11 had moved out of traceable areas or could not be traced (Fig. 1). We also excluded women in the furthest two districts after reaching our IRB-approved convenience sample size of 300 women. During data analysis, we determined that three women did not have an obstetric cause of their fistula, so these women were excluded from the analysis, leaving a final sample size of 297 women with OF.

Most women were enrolled in this long-term follow-up study 24–48 months after their fistula repair (median of 35.5 months [IQR = 27, 43 months]). Of the 297 women enrolled after OF repair, 75 were excluded for being postmenopausal. Of the remaining 222 women, 60 were excluded for prior hysterectomy, and 14 were excluded for prior bilateral tubal ligation. This left 148 women (49.8%) with reproductive potential for the analysis (Table 1). Fifty four (36.5%) of these 148 women had hormone markers drawn at the time of fistula repair. Many of the 148 women with reproductive potential were between 25 and 34 years (45.3%), married (73.7%), and had at least one living child (59.5%) (Table 1). Eighteen (12.2%) were HIV-infected and 117 (79.1%) reported sexual activity within the last month.

**Table 1:** Obstetric and reproductive health characteristics in OF patients with reproductive potential 1–4 years after repair (*n* = 148)

	n (%)
Age	
18–24 years	24 (16.2)
25–34 years	67 (45.3)
35–49 years	57 (38.5)
Relationship status	
Currently married	109 (73.7)
Separated, divorced, or widowed	37 (25.0)
Never married	2 (1.4)
Living children	
0	60 (40.5)
1–2	68 (46.0)
≥ 3	20 (13.5)
HIV Status	
Negative	130 (87.8)
Positive	18 (12.2)
Time of last sexual activity	
< 1 month ago	117 (79.1)
1–12 months ago	10 (6.7)
≥1 year ago	21 (14.2)
Pregnancy since fistula repair	
Yes	30 (20.3)
No	108 (79.7)
Currently menstruating	
Yes	102 (68.9)
No	46 (31.1)
Contraceptive use^a^	
No method	92 (63.9)
Traditional/natural method^b^	3 (2.1)
Modern method^c^	49 (34.0)

^a^If not currently pregnant

^b^Traditional/natural method includes string, chewing peas, using fluids from sticks or leaves, breastfeeding, natural family planning

^c^Modern method includes condoms, pills, injectable, implant, IUD, bilateral tubal ligation

### Reproductive potential and pregnancies since repair

Overall 20.2% of the 148 women reported a pregnancy after fistula repair. Thirty-one pregnancies were reported among 30 women over 5137 person-months since their fistula repair, leading to an incidence rate of 0.60 pregnancies (95% Confidence Interval 0.39, 0.82) per 100 person-months. The 31 pregnancies had the following outcomes: 15 cesarean deliveries, 7 vaginal deliveries, 5 spontaneous abortions, and 4 ongoing pregnancies, for a total of 22 deliveries (Table 2). Of the 30 women who became pregnant since repair, 66.7% reported that they planned to have more children in the future at the time of their OF repair, 73.3% had a stillbirth at the delivery that resulted in their OF, and 56.7% had no living children at the time of OF repair.

**Table 2:** Characteristics of the 30 women and their 31 pregnancies that occurred after OF repair

	n (%)
Outcome of prior delivery that resulted in OF (*n* = 30)	
Live birth	8 (26.7)
Stillbirth	22 (73.3)
No living children at time of OF repair (n = 30)	17 (56.7)
Planned to have more children at time of OF repair (n = 30)	20 (66.7)
Pregnancy outcomes after OF repair (*N* = 31)	
Live birth	21 (67.7)
Stillbirth	1 (3.2)
Spontaneous abortion	5 (16.1)
Ongoing	4 (12.9)
Estimated time between OF repair and conception (N = 31)^a^	
Less than 1 year	9 (29.0)
Between 1 and 2 years	11 (35.5)
Between 2 and 3 years	2 (6.5)
Unknown	11 (35.5)
Mode of delivery (*n* = 22)	7 (31.8)
Vaginal	
Cesarean	15 (68.2)
Place of delivery after OF repair (n = 22)	
Central Hospital (Tertiary level)	4 (18.2)
District/Private/Mission Hospital (Secondary level)	15 (68.2)
Health Center (Primary level)	3 (13.6)
Reported urinary incontinence after most recent delivery (n = 22)	4 (18.2)
Birthweight of infant (n = 22)	
2500–2999 g	5 (22.7)
3000–3999 g	10 (45.5)
4000–4999 g	1 (4.5)
Unknown	6 (27.3)
Status of infant at time of follow-up survey (*n* = 21)	
Alive	20 (95.2)
Deceased (neonatal death)	1 (4.8)

^a^Conception date was estimated by calculating the number of days between fistula repair and delivery and then subtracting 280 days. Time to conception was therefore only estimated among the 22 women who had a vaginal or cesarean section deliveries; estimated conception could not be calculated for the 4 participants who were still pregnant as they had not delivered yet or among the 5 women who had a miscarriage or abortion (no delivery date recorded)

Among the 22 deliveries, 9 (40.9%) were likely conceived within the first year after repair (Table 2), and the estimated median time to conception was 1.07 years (25%, 75% interquartile range: 0.7, 1.32 years). About two-thirds (68.2%) of the women delivered by cesarean. Of note, the largest known infant weight was 4700 g and delivered vaginally, and the mother did not report recurrence of urinary leakage after this delivery. One stillbirth and one neonatal death occurred among these 22 women resulting in a perinatal mortality ([stillbirths and early neonatal deaths]/deliveries) of 9.1%. All deliveries occurred in a health facility, although three out of the seven vaginal deliveries occurred at a health center, which do not have operating theaters. It is unknown why the other four women delivered vaginally when they delivered at a hospital with operative capacity.

Only four (18.2%) of the 22 women with a delivery since repair reported urinary leakage at the time of the follow-up survey after their pregnancy: one who had a vaginal delivery and three who had cesarean deliveries. All four women were between 30 and 40 years of age at the time of the survey. The woman who delivered vaginally (with live infant of unknown infant birthweight) conceived about 7 months after repair of her OF, which was a Goh Type 3BIII. At the time of discharge from her repair, she was noted to have a 1-h pad weight of 9 g and stress urinary incontinence, which persisted at her 3-month and 12-month follow-ups and at the time of this follow-up interview.

The 3 women who delivered by cesarean and had urinary leakage at the time of the follow-up survey had Goh Type 2AIII, 2BIII, and 3CIII fistulas, respectively, and all delivered infants between 3000 and 3200 g. They were all found to be without incontinence at the time of discharge from their OF repair, although one woman was found to have urinary incontinence with sitting and lying down at 1-month follow-up, and a second was found to have a recurrent fistula at her 3-month follow-up. This second woman then had two subsequent fistula repairs and conceived between 6 and 7 months after her third repair. She had reported no urinary leakage at her 3-month follow-up, so her urinary leakage at the time of this survey was new. However, we do not know if her new urinary leakage was a result of recurrent fistula since we could not do an exam in her home. If we conservatively assume that she and the other woman without urinary leakage after OF repair had a recurrent fistula because they labored prior to their cesarean, our fistula recurrence proportion for delivery after successful OF repair would be 9.1% (2 of 22 deliveries).

Ten of the 54 women who had hormone markers drawn at the time of fistula repair reported a pregnancy. The AMH concentrations for these 10 women ranged from 0.212 to 11.276 pg/ml. The median AMH concentration at the time of OF repair for those who became pregnant was 2.83 pg/ml [IQR 1.72, 4.00], which was not significantly different than for those who did not achieve a pregnancy (1.71 pg/ml [IQR 0.57, 3.59] when adjusted for age, *p* = 0.97).

### Amenorrhea

A majority of women with reproductive potential were menstruating at the time of follow-up (*n* = 102, 68.9%). Of those who were not, 25 (59.5%) attributed this to family planning use, 4 (9.5%) were currently pregnant, 7 (15.2%) had recently delivered and/or were breastfeeding, and 10 (21.7%) did not know why they were not having menstrual periods. After excluding women who were currently pregnant, using family planning, or recently delivered/breastfeeding at the time of the long-term follow-up visit, 25 (78.1%) of the 32 women of reproductive potential who were amenorrheic at the time of fistula repair reported resumption of menses.

The median AMH concentration at the time of fistula repair for those whose menses returned was 1.67 pg/ml [IQR 0.20, 2.58], which was not significantly different than for those who remained amenorrheic (1.43 pg/ml [IQR 0.49, 1.76]) after adjustment for age (*p* = 0.23). One of two women who had been diagnosed with hypergonadoptropic hypogonadism (FSH = 25 mIU/ml, estradiol = 15.91 pg/ml) as the cause of her amenorrhea at the time of her fistula repair reported having menses at the time of her follow-up, whereas the other (FSH = 33.5 mIU/ml, estradiol = 16.13 pg/ml) still had amenorrhea. Both women were 42 years old at the time of the follow-up.

### Marriage and sexual activity

Of the 121 women who reported sexual activity in the past 12 months, only 11 reported dyspareunia (8.6%). Dyspareunia was present in women with Goh type 1 (2), Goh type 2 (4), Goh type 3 (3), Goh type 4 (1), and in one women who had an RVF.

### Family planning

Forty-eight women (33.3%) who were not currently pregnant at the time of the long-term follow-up survey reported that they were currently trying to conceive. Three of these women had already reported a pregnancy since OF repair. Of the 96 other women who were not trying to conceive, 51 (53.1%) were currently using a method of family planning. The most common method of family planning used was the implant (*n* = 28, 54.9%), followed by injection (*n* = 7, 13.7%), condoms (n = 7, 13.7%), bilateral tubal ligation (*n* = 6, 11.8%), and natural family planning methods (*n* = 3, 5.9%). Of the remaining 45 women who were not attempting pregnancy or using contraception, 25 (55.6%) had been sexually active in the past year and 20 (44.4%) had not.

## Discussion

In our cohort of 148 women with reproductive potential and prior OF repair, over one-fifth conceived and nearly one-third still desired a pregnancy. Many of these pregnancies resulted in a live birth, and few of the women who became pregnant reported incontinence after delivery. Most women with amenorrhea at the time of repair resumed menses, and few women reported dyspareunia. Approximately half of women not attempting to conceive were using family planning, with implant as the most common method utilized.

A recent review of 11 studies (including one from our study population [4]) found that 212 (17.4%) of 1218 women followed after OF repair became pregnant, with a range of 2.5–40% [17]. Our proportion of 20.2% is well within this range. Possible causes for infertility include ovulatory disorders [7], cervical or uterine scarring [18], vaginal stenosis [11], and not having a sexual partner [1]. However, we are unable to comment on the frequencies of these conditions for the women in this study due to the design of the study using a survey with a non-medical staff person in a survey instead of a clinical exam.

Compared to reports of OF patients in other settings, women in our study who became pregnant after repair had a slightly lower proportion of stillbirth. The previously-mentioned review found that 7.6% of pregnancies ended in stillbirth, whereas our proportion was 4.5%, which is still slightly higher than the 3.4% reported among all pregnancies in Malawi [17, 19]. However, 16.1% of our pregnancies resulted in spontaneous abortion, which is higher than the 5.4% calculated from the review and the 4.7% reported overall in Malawi [20]. In another smaller study of women with and without OF repair in Malawi, of the 10 pregnancies since OF repair, only one resulted in a live birth, and this woman had a recurrence of her fistula [21]. The three largest published series of pregnancies after OF repair reported perinatal mortality proportions of 17–37%; our perinatal mortality of 9.1% was lower than these other studies [21–23].

The differences found in obstetric outcomes between our study and other studies may be related to the differences in population and study design. Unlike other studies, which were retrospective, often without a denominator, and with short follow-up periods, our study was prospective, and we were able to follow-up all eligible women within 8 districts of Malawi up to 4 years after OF repair. Therefore, our study design minimized non-respondent bias, whereas other studies may have oversampled women with either positive or negative reproductive outcomes, depending on the medical behavior patterns in their populations. However, we excluded women who had died since their OF repair since they could not be interviewed. Since we did not know the cause of death for the four women who had died, we were unable to calculate a proportion for maternal death after OF repair.

Only four of 22 women in our study reported urinary incontinence after delivery, and two already had urinary incontinence prior to delivery. Our maximum proportion of 9.1% for fistula recurrence is slightly higher than the 5.0% reported in the review [17] but lower than the 12–33% women reported in other studies [6, 22, 23]. In one of these studies, fistula recurrence was associated with delivery outside the hospital where the repair took place, at home, or after 2 or more days of obstructed labor [22]. In a study of Ethiopian fistula patients, only 59% of women recalled advice to deliver in a hospital after OF repair [5]. Women in our study population received intensive instructions on the recommendation for cesarean delivery with any pregnancies after repair and hence may not be representative of a typical sample of fistula patients. In Malawi, there is no charge for a scheduled cesarean delivery at government hospitals. However, a recent case study from Malawi demonstrated that even when women present to the hospital prior to labor, delay of clinical care can result in perinatal death and fistula recurrence [24].

Amenorrhea is commonly reported in women presenting for OF repair at rates of 22–44% [6, 7, 9]. Possible causes include pituitary gland ischemia from postpartum hemorrhage following obstructed labor (Sheehan’s syndrome) [7], functional hypothalamic amenorrhea from anorexia or depression, or intrauterine scarring (Asherman’s syndrome), but it is often unexplained [18]. A recent analysis that evaluated the causes of amenorrhea among OF patients at the time of repair using FSH, estradiol, and AMH testing and pelvic ultrasound found that 42% were unexplained, 27% were likely from breastfeeding, 12% were from polycystic ovarian syndrome (PCOS), 9% were from use of the depot medroxyprogesterone acetate injectable, 6% were from hypergonadotropic hypogonadism, and 3% were from hypogonadotropic hypogonadism [10].

However, two studies from Nigeria have demonstrated that some women who are amenorrheic at the time of their fistula repair later have return of menses. In both studies, 41% of women resumed menses, within 6–24 months of repair [6, 7]. In our study, we demonstrated that 78% of those who were amenorrheic at the time of repair resumed menses within 1–4 years of fistula repair surgery. Interestingly, one of the women who was determined to have hypergonadotrophic hypogonadism reported resumption of menses. One case study of an adolescent with this condition and galactosemia details a spontaneous resumption of regular menstrual cycles four years after diagnosis with normalization of FSH and LH [25]; otherwise, reports of this condition spontaneously reversing are rare.

We do not have hormonal data on all women, but no difference in the proportion of women reporting a pregnancy or return of menses was seen in comparisons using AMH at the time of fistula repair. However, our sample size was limited. It is possible that AMH is not predictive of these outcomes in the OF population, or we were not able to detect this difference because of the small sample size.

Finally, we found that 51 (34.4%) of our 148 women were currently using a method of family planning. This proportion is lower than the 46.0% for current use of contraception among sexually active women in Malawi and is likely secondary to the fact that 48 (33.3%) of the women in our population were actively trying to conceive [25]. In addition, 28 (18.9%) of our women were using the implant, which is more than double the 9.0% reported among all women in Malawi, whereas only 7 women (4.7%) were using the injectable, compared to 22.5% among all Malawian women [25]. This is likely due to the fact that we trained two of our nurses in implant insertion in November 2013, and this made them more comfortable to counsel women about implants and to provide them at their follow-up visits. In addition, our nurses provide daily morning educational sessions for our patients in the pre-operative and post-operative wards, which includes family planning methods as one of the topics. We recommend that other fistula centers use a similar approach to ensure that they are meeting the needs of women who do not want any future pregnancies or want to delay them.

This study presents reproductive data on one of the largest cohorts of OF patients followed up 1–4 years after fistula repair. By tracing women in their homes, we were able to gather information on women who had not completed any prior clinical follow-up. This led to decreased potential bias arising from follow-up losses. Additionally, we are able to compare reproductive outcomes to reproductive history, physical examination, and hormone marker findings at the time of repair, which has not previously been documented.

However, it is important to note that we relied on participant self-report for pregnancy, menstrual, sexual, and family planning data, which could be prone to error and/or social desirability bias. We did not have access to medical records to verify pregnancy or family planning outcomes due to the geographic distribution of the women’s homes and health care facilities. Additionally, we only recruited and enrolled women living within a predetermined distance of the Fistula Care Centre, which may have created selection bias. Results may not be generalizable to fistula patients in other parts of the world with different access to reproductive services.

## Conclusions

In this long-term follow-up study of women after OF repair, many women were able to achieve a pregnancy with a live birth, have normal menses, resume sexual function, and access contraception. We recommended that other fistula surgeons also ensure that their patients are provided with the comprehensive one-on-one educational counseling that emphasizes the need to wait 1 year before attempting to conceive and to receive appropriate antenatal and delivery care at a hospital for any future pregnancies to minimize fistula recurrence and poor obstetric outcomes. In addition, we recommend that other fistula centers train their staff in all methods of family planning, so that they are comfortable with counseling patients about all options prior to discharge and with providing these methods on site as needed. Further efforts by organizations that care for women with OF should focus on assisting women with achieving their reproductive goals after OF repair, whether that be pregnancy or prevention of unwanted pregnancy. These efforts will greatly assist a population of women for whom reintegration and restoration of dignity is closely tied to their ability to achieve their reproductive goals.

## Abbreviations

AMH: antimüllerian hormone
FSH: follicle-stimulating hormone
HIV: human immunodeficiency virus
LH: luteinizing hormone
OF: Obstetric fistula
